# Clinical and Immunological Efficacy of Mangosteen and Propolis Extracted Complex in Patients with Gingivitis: A Multi-Centered Randomized Controlled Clinical Trial

**DOI:** 10.3390/nu13082604

**Published:** 2021-07-28

**Authors:** Jin-Young Park, Kyung-A Ko, Ji-Yeong Lee, Jae-Woon Oh, Hyun-Chang Lim, Dong-Woon Lee, Seong-Ho Choi, Jae-Kook Cha

**Affiliations:** 1 Department of Periodontology, Research Institute for Periodontal Regeneration, Yonsei University College of Dentistry, Seoul 03722, Korea; jinyoungpark87@gmail.com (J.-Y.P.); kokyunga1064@gmail.com (K.-A.K.); shchoi726@yuhs.ac (S.-H.C.); 2 Innovation Research and Support Center for Dental Science, Yonsei University Dental Hospital, Seoul 03722, Korea; 3 Department of Periodontology, Periodontal-Implant Clinical Research Institute, Kyung Hee University, School of Dentistry, Seoul 02447, Korea; jung2lee.peri@gmail.com (J.-Y.L.); periodent81@gmail.com (H.-C.L.); 4 Department of Periodontology, Dental Hospital, Veterans Health Service Medical Center, Seoul 05368, Korea; ohsoon01@bohun.or.kr (J.-W.O.); dongden@daum.net (D.-W.L.)

**Keywords:** mangosteen, propolis, gingivitis

## Abstract

Background: Mangosteen and propolis extracts (MAEC) have been potential therapeutic agents known to exhibit powerful antioxidant and anti-inflammatory properties. The aim of the current study was to evaluate the clinical and immunological efficacy of MAEC as well as safety and patient-reported outcomes (PROMs) on gingivitis and incipient periodontitis. Methods: This study was performed on 104 patients diagnosed with gingivitis or incipient periodontitis. At baseline, the participants were randomly allocated to either the test group, with daily intake of a single capsule containing 194 mg of MAEC for eight weeks, or control group, with placebo. Clinical periodontal evaluation and immunological parameters from saliva and gingival sulcular fluid were assessed at baseline, four, and eight weeks. Individual PROMs were assessed by OHIP-14 questionnaires. Results: There was a significant difference of modified gingival index at four and eight weeks between the test and control groups. In the test group, crevicular interleukin (IL)-6 was reduced, and the salivary matrix metalloproteinase (MMP)-9 was increased after eight weeks. PROMs were improved up to four weeks compared to placebo. Conclusion: Oral administration of MAEC would have a potential to reduce gingival inflammation clinically and immunologically in the patients with gingivitis and incipient periodontitis.

## 1. Introduction

Gingivitis is an inflammatory condition initiated by the accumulation of dental biofilm and is characterized by gingival swelling and redness [1]. Periodontitis is the more progressive form of inflammation that results in the loss of periodontal attachment and is caused by a multitude of factors, including oral bacteria and host immunity [2]. Often, periodontitis can progress unnoticed, but the eventual loss of teeth is irreversible. Achieving good oral hygiene has been and still is the key to prevention and treatment of these inflammatory disorders [3,4,5]. The standard of care for periodontitis in the professional setting includes mechanical debridement to remove the pathologic biofilm along with the application of antimicrobials. In recent times, research has shown that it is the host’s immunity that is responsible for the progression of inflammation and destruction of periodontium [6]. Therefore, immune-modulation therapies, such as oral bisphosphonates or tetracyclines, have been introduced to achieve the blockage of proinflammatory pathways related to secretion of immune-regulatory cells in the periodontitis niche [7,8], but side-effects or short-comings have been common [6]. Nowadays, the focus on compounds derived from medicinal plants and natural products have increased [9,10]. Safe and efficacious substances extracted from natural sources known to contain antioxidant and anti-inflammatory properties can be taken as daily supplements to reduce the unknown risk of periodontitis.

Mangosteen (*Garcinia mangostana* L.) is an exotic, tropical fruit known for its use in traditional medicine to treat diarrhea, infected wounds, suppuration, and chronic ulcers [10]. The pericarp of mangosteen is a rich source of xanthones, including α-mangostin, β-mangostin, and γ-mangostin. Xanthones have been shown to exhibit various pharmacological properties, including antioxidant, antimicrobial, anti-carcinogenic, and anti-allergic [11]. In particular, α-mangostin compounds exhibit anti-inflammatory activity by inhibiting the production of nitrous oxide, TNF-α, and interleukin-8. A randomized clinical trial using local delivery of 4% mangostana gel into the periodontal pockets of chronic periodontitis patients showed significant improvement in periodontal parameters [12].

Propolis is a viscous substance produced by bees that exhibits antimicrobial, anti-inflammatory, and antioxidant properties [13,14]. Propolis is well-documented in the scientific literature and is known to be non-toxic [15]. Propolis comprises over 200 ingredients, including flavonoids, cinnamic acids, caffeic acids, and caffeic acid phenethyl ester [16]. A meta-analysis on the effectiveness of propolis treatment for periodontitis has shown that it reduced probing pocket depth as compared with placebo [15]. Propolis has been locally delivered in various forms, such as mouthwash, toothpaste, irrigation, and subgingival gel [15], and has been reported to reduce plaque formation and exhibit antimicrobial effects against some key periodontal pathogens [17,18,19]. Furthermore, propolis has been studied extensively for its anti-oxidative property [14], which could be beneficial for treatment of periodontitis, as reduction in oxidative stress may decrease secretion of pro-inflammatory cytokines and prevent alveolar bone loss. One study used propolis as a dietary supplement accompanied by scaling in patients with chronic periodontitis and type 2 diabetes mellitus [20]. Not only was it effective for lowering diabetic biomarkers, such as HbA1c (glycosylated hemoglobin), but also it significantly reduced periodontal parameters.

A recent in vitro study described that the combination of 1:34 ratio in weight of mangosteen and propolis extract were highly effective for reducing IL-6, IL-8, and PGE2 expression in immortalized human fibroblasts treated with lipopolysaccharides (LPS) of *Porphyromonas gingivalis*. Additionally, it was able to induce the most bone forming activity from human osteoblast-like cells. However, there are no clinical studies up to date to verify the efficacy and safety of MAEC [21]. Therefore, the aim of the present randomized controlled trial was to confirm the clinical and immunological outcomes of systemically taken MAEC as well as patient-reported outcomes (PROMs) on patients with gingivitis and incipient periodontitis.

## 2. Materials and Methods

### 2.1. Study Design and Participants

**Figure 1:**
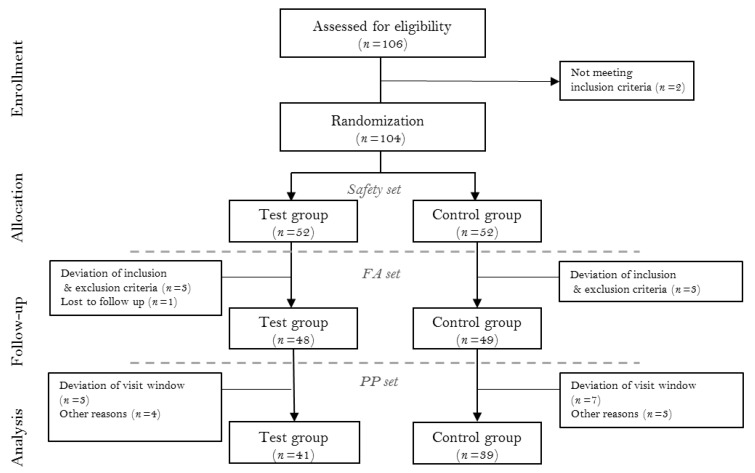
The CONSORT flow chart. FA (full analysis) set, and PP (per protocol) set.

This study was designed as a multi-centered, double-blinded, parallel-armed, placebo-controlled randomized clinical trial. The study was registered with the Clinical Research Information Service of the National Research Institute of Health in the Republic of Korea (KCT0005569). Clinical measurements between investigators were standardized via calibration meetings prior to trial commencement. All procedures were adhering to the Declaration of Helsinki and good clinical practice guidelines and approved by the Institutional Review Board of the Yonsei University Dental Hospital (2-2019-0025), Kyung Hee University Dental Hospital (KH-DT19014), and Veterans Health Service Medical Center (2019-05-018). A total of 104 subjects were enrolled from the Department of Periodontology, Yonsei University Dental Hospital; Department of Periodontology, Kyung Hee University Dental Hospital; and Veterans Health Service Medical Center from September 2019 to March 2020. Before enrollment, all participants were informed about the nature of the study, and informed consent form was obtained. The CONSORT flowchart is presented in Figure 1. The clinical data collection was performed by the resident dentists at the periodontics department of each center.

### 2.2. Inclusion and Exclusion Criteria

This study was performed on patients diagnosed as generalized or localized gingivitis or stage I periodontitis according to the 2017 World Workshop Consensus Report [2]. Further inclusion criteria were (1) being >20 and <75 years of age and in good general health, (2) having a minimum of 18 teeth, (3) having BOP (bleeding on probing) sites of >10%, and (4) having at least one tooth with PD (probing depth) of >3 mm and ≤5 mm. The exclusion criteria were (1) not providing written informed consent; (2) having received preventive periodontal therapy within 3 months prior to screening; (3) having a serious oral mucosal disease such as oral cancer; (4) having >5 carious teeth that require treatment; (5) being diagnosed as chronic moderate or advanced periodontitis; (6) smoking; (7) taken antibiotics in the 3 months prior to screening; (8) having uncontrolled diabetes (fasting blood glucose level > 180 mg/dL); (9) having elevated AST(GOT) or ALT(GPT) levels > 3 times the ULN(upper limit of normal); (10) having creatinine level > twice the ULN; (11) having bleeding disorders or history of hemorrhage and receiving preventive antiplatelet or anticoagulant medication; (12) having a significant cardiovascular, immunological, infectious, or oncological illness, (13); having a mental illness, such as schizophrenia, depression, and drug/alcohol addiction; (14) being allergic to the test substance; (15) being pregnant or lactating; (16) participating in other clinical trials; and (17) judged as being unsuitable for study inclusion by the clinician for some other reason.

### 2.3. Baseline Evaluation

At baseline, clinical examination along with physical examination, measurement of salivary and sulcus biomarkers, and OHIP-14 (oral health impact profile) questionnaire were completed. The OHIP-14 questionnaire was a Korean version that had been validated [22]. Suitability for study inclusion was assessed according to results of examinations from screening and baseline. The included participants were instructed not to receive other therapeutic agents or therapy during the study, including medication or treatment aid for periodontal disease, antiplatelets or anticoagulants, antioxidant vitamins, antibiotics for >1 week, anti-inflammatory drugs, orthodontic treatment or scaling, and mouthwash.

### 2.4. Intervention and Monitoring

At baseline, the participants were randomly allocated to the following groups:

- Test group: Daily intake of a single capsule containing 194 mg of MAEC for 8 weeks (56 days).
- Control group: Placebo capsule without MAEC given in equal administration as the test group.

The dosage and composition of MAEC was determined based on a previous preclinical study. In brief, α-mangostin was extracted from the pericarp of mangosteen fruit and total flavonoids from propolis. The extracted substances were combined at the ratio of 1:34 by weight (mangosteen:propolis). The effective dose in rodents in the previous study was 2.8 mg/kg by weight [23,24]. When converted for an adult human of 70 kg, the dose was approximately 194 mg. The details of the constituents of the test and placebo capsules are shown in Table 1.

No professional oral prophylaxis was provided at the beginning or during the study period, and the participants were instructed to continue with their daily oral hygiene. Scaling was provided at the completion of the study, at week 8. Dietary analysis was performed on each visit, and patients were instructed to avoid regular consumption of foods containing mangosteen and propolis. Nutritional analysis of the obtained data was carried out using an online software (Can 5.0, The Korean Nutrition Society, Seoul, Korea). For immunological analysis, one tooth from each participant displaying the deepest probing depth was chosen and gingival cord was placed in the gingival sulcus for 1 min to collect gingival crevicular fluid (GCF). Saliva was collected by drooling of unstimulated whole saliva. Samples were analyzed using RNA expression and enzyme-linked immunosorbent assay (ELISA). OHIP-14 questionnaire was completed at baseline, 4, and 8 weeks. Safety was monitored by: (i) reporting of any adverse event or drug reaction throughout the study period; (ii) physical examination and vitality tests (blood pressure and pulse) performed at screening, 4, and 8 weeks; and (iii) pathological tests on urine and blood samples performed at screening and 8 weeks. In the reporting of adverse drug reaction, the severity of adverse event was recorded according to the grading system recommended by the World Health Organization [25].

### 2.5. Randomization and Blinding

The participants were randomly allocated to one of two study groups, with fifty patients in each group. Randomization sequence was generated by SAS^®^ system’s (version 9.4, SAS Institute, Cary, NC, USA) randomization program. Sealed envelopes containing manufactured capsules were labeled with unique identification codes that were sent to the clinical centers and sequentially allocated to the enrolled participants on baseline. Identity of the double-blinded samples remained undisclosed until the end of the trial unless the participant was to be excluded.

### 2.6. Clinical Parameters and Immunological Biomarkers for Evaluation of Efficacy

All clinical parameters were recorded on every visit of active treatment. The efficacy of MAEC was evaluated by comparing periodontal indices and immunological indicators at each stage of the trial. The primary outcome of the study was the change in modified gingival index (GI) [26]. Modified GI was recorded at two surfaces of each tooth (buccal and lingual) and every tooth of each patient. The secondary outcomes were changes in PD (probing depth), CAL (clinical attachment loss), PI (plaque index) [27], BOP (bleeding on probing), GR (gingival recession), salivary and crevicular fluid biomarkers (interleukin-1 β (IL-1β), interleukin-6 (IL-6), matrix metalloproteinase-8 (MMP-8), matrix metalloproteinase-9 (MMP-9)), and OHIP-14 (Oral Health Index Profile-14) [28].

### 2.7. Sample Size Estimation

The two-sided test was used to determine the sample size. A previous study having the same evaluation variable as the current study was referred to for calculation [29]. A minimum sample size of 40 patients in each group was needed to detect a clinically relevant difference in the primary outcome measurement between the test and control groups with a statistical power of 80% and a significant level of 5% (SD = 0.5). Considering an estimated dropout rate of 20%, the required sample size was determined to be 100 patients in total.

### 2.8. Statistical Analysis

Statistical analysis was performed with commercially available software SAS^®^ (Version 9.4, SAS Institute). Changes in clinical parameters within the groups between time points were analyzed using either the Paired *t*-test or Wilcoxon signed-rank test according to result of the normality test. Inter-group analysis at each time point was made using the two-sample *t*-test or Wilcoxon rank-sum test. The Mann–Whitney U test and chi-square tests were used to compare demographic characteristics and clinical index between the test and control groups. Smoking, age, sex, alcohol consumption, and obesity were considered as covariates for generalized linear model in efficacy evaluation, as these factors are known to be closely related to the dependent variables. The data were presented in mean and standard deviation, and statistical significance of difference between two groups was defined as *p* < 0.05.

### 2.9. Data Set Characterization

The data collected from the current study were categorized into safety set, FA (full analysis) set, and PP (per protocol) set. The safety set included any participant that ingested the test product at least once. According to the ITT (intention to treat) protocol, FA set included any participant that received the test product at least once, attended at 4 and 8 weeks for efficacy evaluation, and adhered to the inclusion/exclusion criteria. PP set included only the participants that completed the study with adherence to the inclusion criteria. The PP set was used mainly for the evaluation of efficacy, and the FA set analysis was performed additionally. The safety set was used for the safety analysis.

## 3. Results

### 3.1. Participant Flow and Baseline Data

Total 104 patients were enrolled for randomization (52 test, 52 control). Five participants failed to attend after baseline evaluation, and two did not adhere to the inclusion/exclusion criteria. Therefore, 97 participants were included in the FA set. From the FA set, 10 subjects attended outside of the visit window, 3 had taken contraindicated medications, and 4 failed to sign required consent forms. Therefore, 80 subjects completed the study and were included in the PP set (*n* = 41 and 39 for test and control groups, respectively) (Figure 1). Among the baseline characteristics, no statistically significant difference between the test and control groups was found. The baseline demographics and clinical characteristics of each group were presented in Table 2.

### 3.2. Outcomes

#### 3.2.1. Clinical Parameters

The results on clinical parameters for primary and secondary outcomes are summarized in Table 3. Modified GI showed statistically significant reduction in both test and control groups after four (*p* = 0.0002 and 0.004, respectively) and eight weeks (*p* < 0.0001, *p* = 0.004, respectively). The amount of reduction was greater in the test group than control group at both four (*p* = 0.018) and eight weeks (*p* = 0.041). PD, CAL, PI, BOP, and GR showed significant reduction in the test group after eight weeks. However, inter-group analysis showed no significant difference in these parameters between test and control groups.

#### 3.2.2. Immunological Parameters

**Figure 2:**
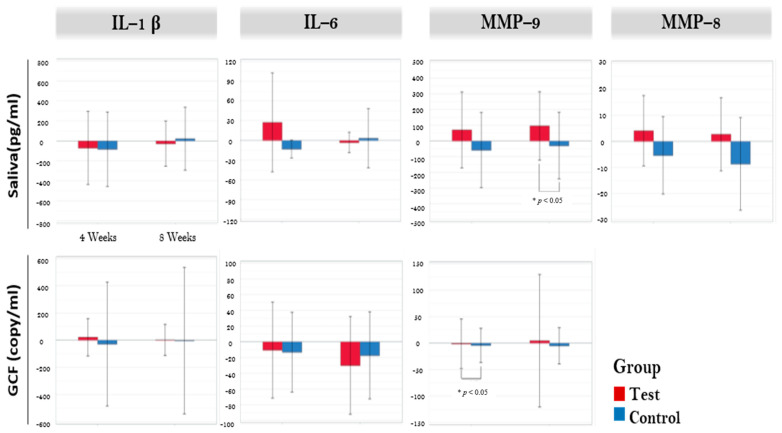
Biomarkers in salivary sample and gingival crevicular fluid. * Significant difference between test and control (*p* < 0.05). GCF: gingival crevicular fluid, IL-1β: Interleukin-1 β, IL-6: interleukin-6, MMP-9: matrix metalloproteinase-9, MMP-8: matrix metalloproteinase-9.

The results on immunological parameters are illustrated in Figure 2. Crevicular IL-6 showed significant reduction in the test group between baseline and eight weeks (*p* = 0.006). However, no inter-group difference was noted in any of the crevicular fluid markers. Salivary MMP-8 was significantly reduced in control group (*p* = 0.022) after eight weeks. Salivary MMP-9 showed significant increase in test group (*p* = 0.041). Inter-group analysis showed significant difference in salivary MMP-9 between the groups (*p* = 0.043). However, when adjusted for covariates (smoke, age, sex, drink, obesity) in the generalized linear model, no statistical significance was found. FA set analysis showed no significant difference in inter-group analysis of all salivary markers. 

#### 3.2.3. Safety Analysis

The safety set data were used to perform the safety analysis (104 in total, 52 in each group). In the test group, eight patients reported nine incidents of mild adverse events, whereas in the control group, eight patients reported ten mild incidents. There was no statistically significant difference between the two groups, and there were no drop-outs among the reported patients. The most frequently reported adverse event was infection or parasitic infection for both test and control groups (5.77% and 7.69%, respectively). Acute gastroenteritis was the most relevant adverse drug reaction among the reported incidents, and it occurred in one patient from the control group. The patient healed spontaneously and completed the study. The clinical parameters for various tests are shown in Table 4. Blood tests revealed that RBC (red blood cell), Hb (hemoglobin), and Hct (hematocrit) were increased in the test group after eight weeks and decreased in control group. These changes were within the limits of normality. Inter-group analysis also showed statistical significance for each of these categories.

#### 3.2.4. Patient-Reported Outcome Measurements (PROMs)

Higher scores from the OHIP-14 questionnaire reflect worse outcome reported by the patients. The results from OHIP-14 were presented on Table 5. The scores from questions regarding functional limitation at four weeks were significantly increased among control group compared to baseline (*p* = 0.034). There was a significant difference between test and control groups at four weeks (*p* = 0.049), which was also evident in the analysis of the FA set. The scores from questions on taste disturbance at four weeks were lower for the test group compared to the control (*p* = 0.01) in the FA set analysis. In response to the question “Have you felt your diet has been unsatisfactory?”, the test group scored significantly lower at both time points compared to baseline (*p* = 0.03 and 0.01 at four and eight weeks, respectively). These scores were significantly lower than for placebo (*p* = 0.02 and 0.03 at four and eight weeks, respectively).

## 4. Discussion

In the present clinical trial, MAEC capsules were orally administered for up to two months in patients with gingivitis and incipient periodontitis and were compared with placebo. The main findings were (i) daily ingestion of MAEC resulted in significant reduction of modified GI at four and eight weeks compared with placebo; (ii) a daily intake of the current dose (194 mg) was safe and without any considerable adverse events; and (iii) MAEC intake significantly improved PROMs compared to placebo.

Modified GI of the test group in the present study decreased significantly at both four-week and eight-week time points and compared to the control group. This was accompanied by reduction in other clinical parameters, including PD, BOP, CAL, and GR after eight weeks, although significant difference to the control group was not demonstrated for these variables. Modified GI is a non-invasive modification of the GI [27] and has been generally accepted for use in clinical trials. Evidence indicates that non-invasive indices, such as the modified GI, are capable of providing comparable data to invasive indices like BOP [30]. Since modified GI expands the low end of the scale of GI to increase sensitivity for assessing gingival inflammation, it was appropriate for detecting subtle changes in the gingival status of patients with mild inflammation, such as in this study.

The present study investigated the effect of MAEC per se without scaling and root planning at baseline. Scaling and root planing disrupts and reduces the gingival and subgingival microbiota [31], which would cause improvement in the clinical parameters. By utilizing the current study design, the focus can be drawn to the influence of MAEC on the host’s response to a chronic bacterial insult. A chronic lesion is one that is characterized by unresolved inflammation, resulting in the loss of tissue structure and function [32]. However, it is still possible that some of the sites of inflammation might be periodic lesions that spontaneously heal with the body’s natural immune system. This was evident in the reduction of modified GI in the placebo group up to eight weeks compared to baseline. Yet, the significant difference in the reduction of modified GI between the test and control groups shows that MAEC would be acting as the catalyst in speeding up the resolution of inflammation.

The results from the immunological parameters in this study were somewhat indicative of the reduction of inflammation. The crevicular IL-6 levels decreased after eight weeks but not significantly compared to the placebo. It was reported that IL-6 was produced by T cells, macrophages, and osteoblasts and was a regulator of T- and B-cell growth and stimulates osteoclast formation [33]. Along with IL-1β, IL-6 in the GCF has been associated with deep PDs and severe gingival inflammation in a sample size of over 6000 patients [34]. The persistent secretion of proinflammatory cytokines, including tumor necrosis factor alpha (TNF-α), IL-1β, IL-6, and IL-12, combined with reduced levels of regulatory cytokines, including IL-10, and transforming growth factor beta 1 (TGF-β1), has been linked with sustained inflammation and loss of periodontal attachment [35]. Therefore, the reduction of IL-6 in the GCF is in correlation with the improvement of the clinical parameters in this study. 

The MMP-9 levels in the saliva were significantly increased in the MAEC group after eight weeks, and this increase was greater than the placebo group. However, when the results were adjusted for covariates, no difference was found. MMP-8 and MMP-9 were chosen for assessment in this study as they are the most common MMPs in periodontal tissues that indicate periodontitis progression, severity, and treatment response [36]. MMPs have been related to a wide spectrum of inflammatory diseases and were traditionally regarded as collagenases and gelatinases that degrade extracellular matrix components [37]. However, further research into MMPs has brought the appreciation of the vast complexity of MMP functions that places them into the present idea of the “protease web” [38]. MMPs have been suggested as the main regulators of inflammation as well as multifunctional proteins, causing pro- or anti-inflammatory reactions that lead to either pathology or homeostasis. In an in vitro study using human fibroblasts, MMP activity reduced the secretion of IL-6 while increasing the secreted levels of some proinflammatory chemokines that activates leukocytes [39], which concurs with the immunological parameters in this study. On the other hand, a meta-analysis involving an excess of 6000 subjects suggested that MMP-9 reduced the risk of periodontitis, whereas MMP-3 and -8 increased the risk of periodontitis [40]. Given that there was a significant reduction of gingival inflammation in the MAEC group with increase in the MMP-9, the current evidence may support the result of that meta-analysis.

The MAEC in this study has been studied previously to determine its anti-inflammatory effect. In addition, the anti-inflammatory effect of MAEC was greater than when mangosteen and propolis extracts were used alone and in combinations with varying concentrations, which suggests that there might be a synergistic effect between the two substances in that specific combination [21]. Since the present study was performed using oral administration, it is unknown whether the same concentration of the mixture was present in the target tissues of the subjects. Although there is abundance of preclinical studies on antioxidant anti-inflammatory properties of mangosteen and propolis, pharmacokinetics of the two substances have received limited attention. A previous study in rodents has revealed that intravenously injected α-mangostin was rapidly distributed and slowly eliminated from blood; however, when orally administered, the bioavailability was low [41]. A more recent study in rodents showed that delivery of xanthones in the form of mangosteen fruit extracts exhibited greater bioavailability of free α-mangostin in serum compared to the delivery in the form of pure xanthones due to slower rate of conjugation [42]. In a human study using oral administration of xanthone-rich fruit juice, xanthones were found to be absorbed and partially conjugated in healthy adults, but there was a high variability in the concentrations found in the plasma and urine [43]. Similarly, for propolis, the number of studies describing the bioavailability is limited. There is great difficulty in determining the absorption and exertion of biological effects through systemic circulation at the desired site of action, which can be affected by numerous factors, such as the food matrix, interactions with other compounds, chemical structure, and concentration [44]. Further in vivo studies are needed to establish the pharmacokinetics of the two substances in order to achieve the desired concentration at the target site.

The dose of MAEC used in this study was safe for oral administration, as there was no difference with the placebo group in terms of the reported incidences, and results from physical examinations and pathologic tests were within the normal range. The lethal dose for rodents was 1000 mg/kg, and the effective dose for anti-tumor activity was found to be between 100 and 200 mg/kg [45], which was much higher than the dose in this study (2.8 mg/kg of MAEC). No adverse events have been reported using mangosteen and propolis extracts separately in previous clinical studies [15,20]. In general, these substances are considered very safe and without side effects, and this study showed that safety was maintained when the two substances were used together.

OHIP-14 questionnaire utilized in this study revealed that the patients experienced improvement in oral health by taking the MAEC capsules daily. OHIP-14 is an abbreviated version of the OHIP-49, a 49-item questionnaire that measures people’s perceptions of the impact of oral conditions on their well-being and has been shown to produce good reliability, validity, and precision [28]. Among the seven domains of the questionnaire, significant outcomes were achieved in the most relevant domains—functional limitation and physical disability, in which the patients experienced improved sense of taste and felt the diet was more satisfactory. It is common knowledge that better dietary function can lead to better overall health and quality of life. In a previous clinical trial, the test group, after taking xanthone-rich mangosteen product, showed significantly enhanced immune responses and improved subject’s self-appraisal on overall health status compared to the placebo [46].

In fact, the blood tests at the end of the study showed that the test group exhibited significantly increased levels of RBC, Hb, and Hct within the limits of normality compared to the placebo group. A recent clinical study involving nearly 1000 subjects revealed that the increases in the levels of Hct, Hb, and RBC within the normal ranges were beneficial for the maintenance of vascular function and structure, which may decrease the risk of cardiovascular disease [47]. Propolis has been extensively studied for its cardiovascular therapeutic potential [48]. A randomized clinical trial on healthy subjects showed that orally administered propolis supplement resulted in a significant decrease in BP after two months [49]. Another double-blinded placebo controlled study demonstrated that oral administration of propolis solution decreased BP in hypertensive patients over 90 days [50]. Positive antioxidant and anti-inflammatory properties of propolis are attributed to caffeic acid phenethyl ester and the total flavonoid content, the latter being the main ingredient of the MAEC in this study. In a study using rodents, animals were subjected to isoproterenol-induced myocardial infarction. Treatment with α-mangostin resulted in alleviation of oxidative changes and lipid peroxidation owing to the antioxidant property of α-mangostin [51]. The results from the present study together with the evidence in the literature suggest that the MAEC may have some added benefits for the cardiovascular system.

Various supplementary substances from natural extracts had been reported previously for reduction of gingival inflammation. For instance, a combination of vitamin C, vitamin E, lysozyme, and carbazochrome showed efficacy for reduction of GI [52]. Green tea extract has been also considered due to the biological activity of its polyphenols namely catechins. This substance was shown to have a therapeutic effect on the damaged periodontal tissue when orally administered [53].

## 5. Conclusions

In summary, in consideration of the significant reduction in modified GI, the MAEC used in this study might exhibit anti-inflammatory potential. The immunological parameters were inconclusive to determine the complex interactions between the MAEC, innate immune system, and the cascade of MMP/cytokine reactions. This data should be interpreted with caution and remain open for discussion in future research. The dose of MAEC was safe for oral administration. Enhanced parameters from blood tests along with PROM can place MAEC in the positive light as a dietary supplement.

## Figures and Tables

**Table 1:** The details of the constituents of the test and placebo capsules.

	Placebo		Test	
Raw Material	Compounding Ratio (%)	Content (mg)	Compounding Ratio (%)	Content (mg)
Mangosteen and propolis extracted complex	-	-	41.28	194.00
Lactose powder	62.70	294.69	30.00	141.00
Microcrystalline cellulose	34.27	161.07	24.22	113.85
Sucrose esters of fatty acids	-	-	2.00	9.40
Magnesium stearate	1.00	4.70	1.50	7.05
silicon dioxide	-	-	1.00	4.70
Caramel color	2.00	9.40	-	-
Food blue No.1	0.03	0.14	-	-
Total	100	470	100	470

**Table 2:** Demographics and clinical characteristics of participants.

Variables	Control Group (*n* = 39)	Test Group (*n* = 41)	*p*-Value
Age (years), mean ± SD	33.41 ± 6.89	35.95 ± 10.64	0.4520 ^†^
Gender, *n* (%)			
Male	7 (17.95)	12 (29.27)	0.2344 ^#^
Female	32 (82.05)	29 (70.73)	
Smoking status, *n* (%)			
No	39 (100.00)	41 (100.00)	-
Yes	0 (0.00)	0 (0.00)	
Physical activity, *n* (%)			
None	11 (28.21)	13 (31.71)	0.1057 ^‡^
1–2 times/week	10 (25.64)	8 (19.51)	
3 times/week	15 (38.46)	8 (19.51)	
4–5 times/week	2 (5.13)	7 (17.07)	
7 times/week	1 (2.56)	5 (12.20)	
Drinking status, *n* (%)			
None	24 (61.54)	17 (41.46)	0.3504 ^‡^
Quit drinker	0 (0.00)	1 (2.44)	
Light drinker	7 (17.95)	13 (31.71)	
Moderate drinker	6 (15.38)	7 (17.07)	
Heavy drinker	2 (5.13)	3 (7.32)	
Dental treatment within 3 months, *n* (%)			
No	38 (97.44)	41 (100.00)	0.4875 ^‡^
Yes	1 (2.56)	0 (0.00)	
Height (cm), mean ± SD	165.58 ± 6.44	164.43 ± 9.55	0.2052 ^#^
Number of natural teeth, mean ± SD	26.69 ± 1.94	27.56 ± 1.82	0.0654 ^#^

^#^ Obtained from Wilcoxon rank-sum test, ^†^ obtained from chi-square test, ^‡^ obtained from Fisher’s exact test.

**Table 3:** Clinical parameters of test and control group (mean ± SD).

Parameters	Study Group	Baseline ^†^	4 Weeks ^‡^	8 Weeks ^‡^
PD	Control	2.46 ± 0.25	2.42 ± 0.26	2.42 ± 0.24
	Test	2.58 ± 0.27	2.57 ± 0.27	2.46 ± 0.34
	*p*-value (Test vs. Control)	0.0618	0.1761	0.7879
CAL	Control	2.57 ± 0.26	2.53 ± 0.24	2.48 ± 0.25
	Test	2.67 ± 0.27	2.64 ± 0.29	2.54 ± 0.28
	*p*-value (Test vs. Control)	0.0743	0.5005	0.5940
BOP	Control	41.12 ± 11.20	34.44 ± 16.83	27.00 ± 13.90
	Test	42.56 ± 12.76	31.31 ± 16.95	27.65 ± 19.22
	*p*-value (Test vs. Control)	0.6371	0.1231	0.7272
GR	Control	0.38 ± 0.19	0.28 ± 0.21	0.24 ± 0.19
	Test	0.31 ± 0.15	0.22 ± 0.21	0.23 ± 0.19
	*p*-value (Test vs. Control)	0.1891	0.9078	0.5763
MGI	Control	0.79 ± 0.22	0.66 ± 0.46	0.59 ± 0.53
	Test	0.84 ± 0.33	0.58 ± 0.46	0.50 ± 0.51
	*p*-value (Test vs. Control)	0.8884	0.0184 *	0.0406 *
PI	Control	0.91 ± 0.31	0.78 ± 0.49	0.78 ± 0.40
	Test	0.93 ± 0.33	0.78 ± 0.48	0.66 ± 0.46
	*p*-value (Test vs. Control)	0.9374	0.9143	0.1354

* Statistically significant correlation at *p* < 0.05. ^†^ Obtained from Wilcoxon rank-sum test. **^‡^** Obtained from generalized linear model adjusted baseline, smoke, age, sex, drink, obesity. PD, probing depth; CAL, clinical attachment loss; BOP, bleeding on probing; GR, gingival recession; MGI, modified gingival index; PI; plaque index.

**Table 4:** Safety analysis of control and test at baseline, 8 weeks (mean ± SD).

			Control (*n* = 52)		Test (*n* = 52)	*p*-Value
		*n*	Mean ± SD	*n*	Mean ± SD	
WBC (103/μL)	Baseline	52	6.37 ± 1.50	52	5.91 ± 1.48	0.0885 ^#^
	8 weeks	50	6.16 ± 1.42	48	6.03 ± 1.65	0.1850 ^#^
	*p-*value		0.2574 **		0.3750 ^@^	
RBC (106/μL)	Baseline	52	4.48 ± 0.39	52	4.55 ± 0.43	0.7158 ^#^
	8 weeks	50	4.48 ± 0.38	48	4.61 ± 0.40	0.0483 *
	*p-*value **		0.8040		0.0101	
Hb (g/dL)	Baseline	52	13.50 ± 1.05	52	13.94 ± 1.22	0.1361 ^#^
	8 weeks	50	13.54 ± 1.06	48	14.18 ± 1.18	0.0381 *
	*p* value **		0.8624		0.0020	
Hct (%)	Control	52	40.27 ± 3.04	52	41.06 ± 3.28	0.3907 ^#^
	Test	50	40.30 ± 3.07	48	41.83 ± 3.39	0.0120 *
	*p-*value **		0.8583		0.0003	
PLT (103/μL)	Baseline	52	255.69 ± 54.75	52	269.40 ± 54.23	0.1340 ^#^
	8 weeks	50	253.74 ± 52.71	48	268.27 ± 54.60	0.8496 *
	*p-*value **		0.4001		0.3227	
AST(GOT) (IU/L)	Baseline	52	19.38 ± 7.95	52	21.00 ± 8.81	0.0356 ^#^
	8 weeks	50	20.18 ± 10.53	48	21.06 ± 8.38	0.9517 ^#^
	*p*-value		0.9147 ^@^		0.7794 **	
ALT(GPT) (IU/L) ^@^	Baseline	52	16.23 ± 9.02	52	19.81 ± 16.81	0.1501 ^#^
	8 weeks	50	18.36 ± 13.04	48	18.85 ± 14.80	0.1332 ^#^
	*p*-value				0.3130	
Glucose (mg/dL) ^@^	Baseline	52	94.69 ± 12.52	52	97.56 ± 17.03	0.5195 ^#^
	8 weeks	50	95.56 ± 12.69	48	95.00 ± 11.16	0.2431 ^#^
	*p*-value		0.3297		0.4144	
BUN (mg/dL)	Baseline	52	12.27 ± 3.15	52	12.78 ± 3.58	0.6419 ^#^
	8 weeks	50	11.88 ± 2.77	48	12.44 ± 3.42	0.8643 *
	*p*-value **		0.3753		0.5011	
Creatinine (mg/dL)	Baseline	52	0.74 ± 0.13	52	0.77 ± 0.16	0.5715 ^#^
	8 weeks	50	0.73 ± 0.14	48	0.76 ± 0.15	0.8625 *
	*p-*value **		0.5020		0.6074	

Boldface denotes statistical significance (*p* < 0.05). * Compared between groups; *p*-value for Two sample *t*-test, ^#^ compared between groups; *p*-value for Wilcoxon rank-sum test, ** compared within group; *p*-value for paired *t*-test, ^@^ compared within group; *p*-value for Wilcoxon signed-rank test. WBC, white blood cell; RBC, red blood cell; Hb, hemoglobin; Hct, hematocrit; PLT, platelet; AST, aspartate aminotransferase; ALT, alanine transaminase; BUN, blood urea nitrogen.

**Table 5:** Patient-reported outcome measurements.

			Control *n* = 49	Test *n* = 48	*p*-Value ^#^	*p*-Value ^$^
			Mean ± SD	Mean ± SD		
Functional limitation	Trouble pronouncing words	Baseline	1.33 ± 0.63	1.27 ± 0.49	0.8237	
		4 weeks	1.43 ± 0.65	1.23 ± 0.63	0.2088	0.2100
		*p*-value ^@^	0.2830	0.7813		
		8 weeks	1.24 ± 0.43	1.19 ± 0.49	0.5968	0.7856
		*p*-value ^@^	0.6334	0.3984		
	Worsened sense of taste	Baseline	1.16 ± 0.43	1.17 ± 0.43	0.9717	
		4 weeks	1.22 ± 0.42	1.06 ± 0.24	0.0212	0.0173
		*p*-value ^@^	0.3750	0.1250		
		8 weeks	1.22 ± 0.42	1.10 ± 0.31	0.1371	0.1229
		*p*-value ^@^	0.5488	0.3750		
Physical pain	Painful aching	Baseline	1.45 ± 0.74	1.50 ± 0.68	0.5463	
		4 weeks	1.45 ± 0.68	1.33 ± 0.63	0.1709	0.3582
		*p*-value ^@^	1.0000	0.1668		
		8 weeks	1.39 ± 0.67	1.31 ± 0.59	0.2082	0.5073
		*p*-value ^@^	0.6334	0.0930		
	Uncomfortable to eat	Baseline	1.53 ± 0.79	1.54 ± 0.71	0.7229	
		4 weeks	1.55 ± 0.77	1.38 ± 0.64	0.0880	0.1439
		*p*-value ^@^	0.9744	0.1710		
		8 weeks	1.35 ± 0.56	1.25 ± 0.53	0.4344	0.6614
		*p*-value ^@^	0.1422	0.0048		
Psychological discomfort	Self-conscious	Baseline	1.65 ± 1.01	1.58 ± 0.82	1.0000	
		4 weeks	1.53 ± 0.77	1.35 ± 0.73	0.3905	0.4626
		*p*-value ^@^	0.2970	0.0986		
		8 weeks	1.35 ± 0.63	1.35 ± 0.70	0.5869	0.4328
		*p*-value ^@^	0.0178	0.1108		
	Felt nervous	Baseline	1.80 ± 0.91	1.83 ± 0.97	0.9126	
		4 weeks	1.80 ± 0.91	1.60 ± 0.84	0.1712	0.2543
		*p*-value ^@^	0.9073	0.0467		
		8 weeks	1.69 ± 0.87	1.42 ± 0.71	0.2012	0.0997
		*p*-value ^@^	0.3907	0.0037		
Physical disability	Diet has been unsatisfactory	Baseline	1.43 ± 0.68	1.56 ± 0.68	0.2451	
		4 weeks	1.47 ± 0.62	1.33 ± 0.60	0.0214	0.1167
		*p*-value ^@^	0.7539	0.0340		
		8 weeks	1.49 ± 0.71	1.29 ± 0.50	0.0377	0.1518
		*p*-value^@^	0.5797	0.0124		
	Interrupted meals	Baseline	1.47 ± 0.84	1.44 ± 0.71	0.9717	
		4 weeks	1.33 ± 0.52	1.31 ± 0.62	0.7604	0.9048
		*p*-value ^@^	0.1975	0.3150		
		8 weeks	1.29 ± 0.54	1.25 ± 0.56	0.7977	0.8772
		*p*-value ^@^	0.1715	0.0571		
Psychological disability	Difficult to relax	Baseline	1.78 ± 0.87	1.88 ± 0.96	0.6722	
		4 weeks	1.84 ± 0.96	1.58 ± 0.92	0.0813	0.0629
		*p*-value ^@^	0.7842	0.0032		
		8 weeks	1.61 ± 0.79	1.48 ± 0.71	0.1015	0.2839
		*p*-value ^@^	0.1865	0.0046		
	Embarrassment	Baseline	1.61 ± 0.81	1.50 ± 0.68	0.5996	
		4 weeks	1.55 ± 0.84	1.29 ± 0.50	0.5498	0.2202
		*p*-value ^@^	0.7129	0.0458	0.6297	0.5198
		8 weeks	1.31 ± 0.55	1.17 ± 0.38		
		*p*-value ^@^	0.0209	0.0007		
Social disability	Irritable with other people	Baseline	1.82 ± 0.88	1.81 ± 0.89	0.9690	
		4 weeks	1.94 ± 1.09	1.77 ± 0.88	0.3775	0.4092
		*p*-value ^@^	0.4119	0.8257		
		8 weeks	1.65 ± 0.88	1.52 ± 0.74	0.5813	0.6269
		*p*-value ^@^	0.2173	0.0239		
	Difficulty doing usual jobs	Baseline	1.47 ± 0.71	1.44 ± 0.68	0.8563	
		4 weeks	1.61 ± 0.81	1.40 ± 0.68	0.2417	0.3442
		*p*-value ^@^	0.2523	0.8074		
		8 weeks	1.37 ± 0.64	1.21 ± 0.50	0.2329	0.2243
		*p*-value ^@^	0.3635	0.0510		
Handicap	Less satisfaction	Baseline	1.55 ± 0.77	1.50 ± 0.74	0.6916	
		4 weeks	1.67 ± 0.88	1.52 ± 0.71	0.6577	0.7531
		*p*-value ^@^	0.1855	0.9542		
		8 weeks	1.35 ± 0.66	1.35 ± 0.56	0.7046	0.4157
		*p*-value ^@^	0.0861	0.1185		
	Unable to function	Baseline	1.22 ± 0.42	1.10 ± 0.31	0.1135	
		4 weeks	1.18 ± 0.49	1.13 ± 0.33	0.4418	0.8228
		*p*-value ^@^	0.7539	1.0000		
		8 weeks	1.12 ± 0.33	1.13 ± 0.33	0.1312	0.3290
		*p*-value ^@^	0.2266	1.0000		

^#^: Compared between groups; *p*-value for Wilcoxon rank-sum test; ^$^ Compared between groups; *p*-value for GLM-adjusted baseline, smoke, age, sex, drink, obesity; ^@^ Compared within group; *p*-value for Wilcoxon signed-rank test.

## Data Availability

The data presented in this study are available on request from the corresponding author.
